# Supplementary material for: Probiotic Supplementation Enhances the Effects of a Nutritional Intervention on Quality of Life in Women with Hashimoto’s Thyroiditis—A Double-Blind Randomised Study
Source: Nutrients. 2025 Oct 28;17(21):3387. doi: 10.3390/nu17213387 (PMC12609251; doi:10.3390/nu17213387)
Supplement: Supplementary file 1 [file nutrients-17-03387-s001.zip › nutrients-3924316-supplementary.pdf]

Table S1. *Body mass status and health risk parameters at the baseline and after the intervention among studied women (n=64).*

Parameters	NE+*Lp299v* group N (%)	NE+P group N (%)	P- value between groups[1]
**Body weight status based on BMI**			
Baseline			
Normal body weight	6 (18.7)	12 (37.5)	0.095
Overweight or obesity	26 (81.2)	20 (62.5)	
Post intervention			
Normal body weight	8 (25.0)	13 (40.6)	0.183
Overweight or obesity	24 (75.0)	19 (59.4)	
Baseline vs. post intervention (p - value)[2]	0.004	0.296	
**Waist circumference - metabolic risk**			
Baseline			
Small/normal (≤ 80 cm)	4 (12.5)	7 (21.8)	0.523
Increased (>80-88 cm)	6 (18.7)	7 (21.8)	
Substantially increased (>88 cm)	22 (68.7)	18 (56.2)	
Post intervention			
Small/normal (≤ 80 cm)	4 (12.5)	9 (28.1)	0.298
Increased (>80-88 cm)	5 (15.6)	4 (12.5)	
Substantially increased (>88 cm)	23 (71.9)	19 (59.4)	
Baseline vs. post intervention (p - value)[1]	0.955	0.575	
**WHR**			
Baseline			
No abdominal obesity (< 0.80)	10 (31.3)	10 (31.3)	N/A
Abdominal obesity (≥ 0.80)	22 (68.7)	22 (68.7)	
Post intervention			
No abdominal obesity (< 0.80)	5 (15.6)	10 (31.3)	0.140
Abdominal obesity (≥ 0.80)	27 (84.4)	22 (68.7)	
Baseline vs. post intervention (p - value)[2]	0.002	N/A	
**WHtR**			
Baseline			
No increased risk (< 0.5)	5 (15.6)	8 (25.0)	0.582
Increased risk (0.5 - 0.59)	14 (43.7)	14 (43.7)	
Very high risk (≥ 0.6)	13 (40.6)	10 (31.2)	
Post intervention			
No increased risk (< 0.5)	5 (15.6)	9 (28.1)	0.150
Increased risk (0.5 - 0.59)	13 (40.6)	16 (50.0)	
Very high risk (≥ 0.6)	14 (43.7)	7 (21.9)	
Baseline vs. post intervention (p - value)[1]	0.964	0.696	

[1] – the chi-square test; [2] - the McNemar test; BMI – body mass index; WHR – waist-to-hip ratio; WHtR - waist-to-height ratio; N/A - not applicable.

Table S2. Diet quality indicators among studied women (n=64).

Parameters	NE+*Lp299v* group N (%)	NE+P group N (%)	P- value between groups[1]
**pHDI**			
Baseline			
Low	24 (75.0)	25 (78.1)	0.768
Moderate	8 (25.0)	7 (21.9)	
Post intervention			
Low	22 (68.7)	23 (71.9)	0.784
Moderate	10 (31.2)	9 (28.1)	
Baseline vs. post intervention (p - value)[2]	0.018	0.006	
**nHDI**			
Baseline			
Low	32 (100)	32 (100)	N/A
Moderate	0 (0)	0 (0)	
Post intervention			
Low	32 (100)	32 (100)	N/A
Moderate	0 (0)	0 (0)	
Baseline vs. post intervention (p - value)[2]	N/A	N/A	
**DQI**			
Baseline			
Low intensity of unhealthy and health-promoting traits	28 (87.5)	31 (96.9)	0.162
High intensity of health-promoting traits	4 (12.5)	1 (3.1)	
Post intervention			
Low intensity of unhealthy and health-promoting traits	24 (75.0)	25 (78.1)	0.768
High intensity of health-promoting traits	8 (25.0)	7 (21.9)	
Baseline vs. post intervention (p - value)[2]	<0.001	<0.001	

[1] – the chi-square test ; [2] - the McNemar test; pHDI – the Prohealthy-Diet-Index; nHDI – the Non-Healthy Diet Index; DQI – the Diet Quality Index; N/A - not applicable.

Table S3. The frequency of consumption of selected product groups (times per day) among the studied women (SD, standard deviation; Me, median).

Parameters	Baseline Me (Q1 – Q3)		P- value between groups[1]
	NE+*Lp299v* group N=32	NE+P group N=32	
**pHDI-10 index (products)**			
Wholemeal bread			
Baseline	0.48 ± 0.59 (0.32)	0.39 ± 0.45 (0.14)	0.897
After the intervention	0.73 ± 0.69 (0.50)	0.52 ± 0.48 (0.50)	0.382
Baseline vs. post intervention (p - value)[2]	0.022	0.156	
Buckwheat. oatmeal. whole grain pasta			
Baseline	0.36 ± 0.46 (0.14)	0.33 ± 0.31 (0.14)	0.345
After the intervention	0.35 ± 0.34 (0.14)	0.48 ± 0.28 (0.50)	0.040
Baseline vs. post intervention (p - value)[2]	0.948	0.045	
Milk			
Baseline	0.86 ± 0.77 (1.00)	0.87 ± 0.75 (0.75)	0.789
After the intervention	0.66 ± 0.69 (0.50)	0.73 ± 0.73 (0.50)	0.749
Baseline vs. post intervention (p - value)[2]	0.053	0.132	
Fermented milk drinks			
Baseline	0.34 ± 0.30 (0.32)	0.41 ± 0.43 (0.32)	0.685
After the intervention	0.36 ± 0.29 (0.50)	0.33 ± 0.27 (0.32)	0.782
Baseline vs. post intervention (p - value)[2]	0.570	0.472	
Cottage cheese			
Baseline	0.21 ± 0.20 (0.14)	0.23 ± 0.27 (0.10)	0.662
After the intervention	0.22 ± 0.22 (0.06)	0.21 ± 0.22 (0.06)	0.656
Baseline vs. post intervention (p - value)[2]	0.938	0.372	
White meat			
Baseline	0.36 ± 0.24 (0.50)	0.38 ± 0.23 (0.50)	0.710
After the intervention	0.43 ± 0.23 (0.50)	0.38 ± 0.23 (0.50)	0.341
Baseline vs. post intervention (p - value)[2]	0.094	0.917	
Fish			
Baseline	0.12 ± 0.13 (0.06)	0.13 ± 0.15 (0.06)	0.711
After the intervention	0.20 ± 0.18 (0.14)	0.23 ± 0.21 (0.14)	0.983
Baseline vs. post intervention (p - value)[2]	0.013	0.012	
Legume seed dishes			
Baseline	0.13 ± 0.17 (0.06)	0.12 ± 0.13 (0.06)	0.477
After the intervention	0.20 ± 0.36 (0.06)	0.22 ± 0.39 (0.06)	0.759
Baseline vs. post intervention (p - value)[2]	0.098	0.266	
Fruit			
Baseline	0.89 ± 0.59 (1.00)	0.88 ± 0.61 (0.50)	0.741
After the intervention	0.95 ± 0.61 (1.00)	0.90 ± 0.57 (1.00)	0.717
Baseline vs. post intervention (p - value)[2]	0.587	0.972	
Vegetables			
Baseline	1.33 ± 0.71 (1.50)	1.50 ± 0.64 (2.00)	0.320
After the intervention	1.58 ± 0.61 (2.00)	1.61 ± 0.61 (2.00)	0.833
Baseline vs. post intervention (p - value)[2]	0.069	0.460	
**nHDI-14 (products)**			
White bread			
Baseline	0.56 ± 0.64 (0.50)	0.62 ± 0.69 (0.50)	0.689
After the intervention	0.42 ± 0.46 (0.32)	0.45 ± 0.55 (0.50)	0.961
Baseline vs. post intervention (p - value)[2]	0.288	0.069	
White rice, pasta, small groats			

Baseline	0.27 ± 0.26 (0.10)	0.28 ± 0.25 (0.14)	0.681
After the intervention	0.20 ± 0.22 (0.14)	0.25 ± 0.31 (0.14)	0.840
Baseline vs. post intervention (p - value)²	0.227	0.457	
Fast food			
Baseline	0.10 ± 0.14 (0.06)	0.07 ± 0.04 (0.06)	0.858
After the intervention	0.06 ± 0.04 (0.06)	0.07 ± 0.08 (0.06)	0.622
Baseline vs. post intervention (p - value)²	0.037	0.263	
Fried foods			
Baseline	0.27 ± 0.22 (0.14)	0.36 ± 0.40 (0.14)	0.353
After the intervention	0.22 ± 0.23 (0.14)	0.17 ± 0.17 (0.14)	0.499
Baseline vs. post intervention (p - value)²	0.323	0.003	
Butter as an addition to dishes			
Baseline	0.03 ± 0.09 (0.00)	0.00 ± 0.01 (0.00)	0.874
After the intervention	0.08 ± 0.21 (0.00)	0.06 ± 0.35 (0.00)	0.311
Baseline vs. post intervention (p - value)²	0.101	0.003	
Lard			
Baseline	0.03 ± 0.09 (0.00)	0.00 ± 0.01 (0.00)	0.046
After the intervention	0.08 ± 0.21 (0.00)	0.06 ± 0.35 (0.00)	0.005
Baseline vs. post intervention (p - value)²	0.255	0.655	
Cheeses			
Baseline	0.41 ± 0.43 (0.32)	0.38 ± 0.32 (0.50)	0.961
After the intervention	0.27 ± 0.21 (0.14)	0.29 ± 0.37 (0.14)	0.672
Baseline vs. post intervention (p - value)²	0.032	0.052	
Cold cuts, sausages			
Baseline	0.63 ± 0.56 (0.50)	0.53 ± 0.63 (0.50)	0.161
After the intervention	0.42 ± 0.33 (0.50)	0.28 ± 0.40 (0.06)	0.043
Baseline vs. post intervention (p - value)²	0.025	0.001	
Red meat			
Baseline	0.24 ± 0.21 (0.14)	0.24 ± 0.40 (0.06)	0.190
After the intervention	0.25 ± 0.20 (0.14)	0.17 ± 0.18 (0.06)	0.077
Baseline vs. post intervention (p - value)²	0.753	0.363	
Sweets			
Baseline	0.65 ± 0.53 (0.50)	0.51 ± 0.41 (0.50)	0.313
After the intervention	0.28 ± 0.31 (0.06)	0.33 ± 0.40 (0.14)	0.338
Baseline vs. post intervention (p - value)²	<0.001	0.015	
Canned meat			
Baseline	0.01 ± 0.03 (0.00)	0.02 ± 0.03 (0.00)	0.777
After the intervention	0.01 ± 0.02 (0.00)	0.01 ± 0.02 (0.00)	0.750
Baseline vs. post intervention (p - value)²	0.686	0.225	
Sweetened carbonated or non-carbonated beverages			
Baseline	0.17 ± 0.38 (0.06)	0.19 ± 0.40 (0.06)	0.841
After the intervention	0.13 ± 0.23 (0.06)	0.11 ± 0.22 (0.06)	0.687
Baseline vs. post intervention (p - value)²	0.333	0.041	
Energy drinks			
Baseline	0.02 ± 0.04 (0.00)	0.03 ± 0.12 (0.00)	0.345
After the intervention	0.04 ± 0.10 (0.00)	0.03 ± 0.09 (0.00)	0.334
Baseline vs. post intervention (p - value)²	0.106	1.000	
Alcoholic			
Baseline	0.13 ± 0.17 (0.06)	0.10 ± 0.09 (0.06)	0.889
After the intervention	0.07 ± 0.09 (0.06)	0.09 ± 0.09 (0.06)	0.225
Baseline vs. post intervention (p - value)²	0.028	0.910	

**Other products (FFQ-6 questionnaire)**			
Cruciferous vegetables			
Baseline	0.17 ± 0.22 (0.10)	0.20 ± 0.25 (0.10)	0.400
After the intervention	0.27 ± 0.35 (0.10)	0.28 ± 0.29 (0.10)	0.393
Baseline vs. post intervention (p - value)[2]	0.223	0.187	
Tomatoes			
Baseline	0.21 ± 0.27 (0.06)	0.86 ± 0.47 (1.00)	<0.001
After the intervention	0.70 ± 0.48 (0.57)	0.73 ± 0.36 (0.57)	0.562
Baseline vs. post intervention (p - value)[2]	<0.001	0.196	

[1] – the Mann-Whitney test; [2] – the Wilcoxon test; pHDI – the Prohealthy-Diet-Index; nHDI – the Non-Healthy Diet Index; FFQ-6 – the Food Frequency Questionnaire.

Table S4. The percentage of compliance with the Polish standards according to NIZP-PZH 2020 for vitamins and minerals (n=49).

Parameters	NE+*Lp299v* group N=26 N (%)	NE+P group N=23 N (%)	P- value between groups[1]
**Sodium***			
Baseline	10 (38.5)	8 (34.8)	0.790
Post intervention	12 (46.1)	9 (39.1)	0.620
Baseline vs. post intervention (p - value)[2]	0.571	0.307	
**Calcium**			
Baseline	11 (42.3)	13 (56.5)	0.320
Post intervention	10 (38.5)	12 (52.2)	0.335
Baseline vs. post intervention (p - value)[2]	0.424	0.831	
**Magnesium**			
Baseline	23 (88.5)	21 (91.3)	0.743
Post intervention	22 (84.6)	22 (95.7)	0.203
Baseline vs. post intervention (p - value)[2]	0.003	<0.001	
**Iron**			
Baseline	26 (100)	23 (100)	N/A
Post intervention	26 (100)	22 (95.7)	0.283
Baseline vs. post intervention (p - value)[2]	N/A	<0.001	
**Zinc**			
Baseline	24 (92.3)	22 (95.7)	0.626
Post intervention	26 (100)	22 (95.7)	0.283
Baseline vs. post intervention (p - value)[2]	<0.001	N/A	
**Iodine**			
Baseline	4 (15.4)	5 (21.7)	0.566
Post intervention	3 (11.5)	6 (26.1)	0.189
Baseline vs. post intervention (p - value)[2]	<0.001	0.025	
**Vitamin D (diet + supplementation)**			
Baseline	13 (50.0)	15 (65.2)	0.283
Post intervention	19 (73.1)	20 (87.0)	0.229
Baseline vs. post intervention (p - value)[2]	0.377	0.038	
**Folates (vit. B_9)**			
Baseline	18 (69.2)	18 (78.3)	0.475
Post intervention	20 (76.9)	16 (69.6)	0.560
Baseline vs. post intervention (p - value)[2]	0.038	0.029	
**Vitamin B_{12}**			
Baseline	23 (88.5)	23 (100)	0.093
Post intervention	25 (96.1)	19 (82.6)	0.118
Baseline vs. post intervention (p - value)[2]	<0.001	<0.001	

[1] – the chi-square test; [2] – the McNemar test; N/A - not applicable; *percentage of individuals who did not exceeded the dietary sodium intake according to UL (the Tolerable Upper Intake Level), i.e. 2000mg
